# Supplementary material for: AffyMAPSDetector: a software tool to characterize Affymetrix GeneChip™ expression arrays with respect to SNPs
Source: BMC Bioinformatics. 2007 Jul 30;8:276. doi: 10.1186/1471-2105-8-276 (PMC1959249; doi:10.1186/1471-2105-8-276)
Supplement: Additional file 13 — AffyMAPSDetector v1 source code. AffyMAPSDetector source code. [file 1471-2105-8-276-S13.zip › AffyMAPSDetector_v1_src/doc/affychip/snpdetector/model/OptimizedSnpDetector.html]

OptimizedSnpDetector


|  |  |  |  |
| --- | --- | --- | --- |
| **Overview** \| **Package** \| **Class** \| **Use** \| **Tree** \| **Deprecated** \| **Index** \| **Help** | | |  |
| **PREV CLASS**   **NEXT CLASS** | **FRAMES**    **NO FRAMES**     **All Classes** |  |  |
| SUMMARY: NESTED \| FIELD \| CONSTR \| METHOD | DETAIL: FIELD \| CONSTR \| METHOD |  |  |


---


## affychip.snpdetector.model Class OptimizedSnpDetector

```
java.lang.Object
  affychip.snpdetector.model.SnpDetector
      affychip.snpdetector.model.OptimizedSnpDetector
```

---

public class **OptimizedSnpDetector** extends SnpDetector

Title: Probe SNP Detector

Extension of `SnpDetector` that uses eUtils to find SNPs in a
given GenBank/RefSeq Acc No (Acc=Accession, No=number). Note that with
eUtils, we don't really have to use LocusLink information (GenBank/RefSeq
would suffice). Also, note that SNPs may or may not be annotated on
GenBank records depending upon what the submitter included. RefSeqs are
annotated with SNPs by NCBI and by far the bulk of SNP annotations are
found on RefSeqs as a result of NCBI's annotation.

So, this class first tries to find a RefSeq corresponding to a given
GenBank accession number. Here the strategy used is:

1. Do eSearch on nucleotide database with term=<GenBank/RefSeq Acc No>
2. If the Acc No is not a RefSeq, then use Id returned in eSearch to link
   nucleotide database to itself with
   term=srcdb+refseq[prop]+AND+biomol+mrna[prop]+AND+human[orgn]. Pick the top
   RefSeq id retunred by the query.
3. Do eFetch by using the RefSeq id obtained in step 1 or 2.

Copyright: Copyright (c) 2004

Company: George Mason University

**Version:**
:   1.1

**Author:**
:   Sunita Kumari

---

|  |  |
| --- | --- |
| **Field Summary** | |
| `static java.lang.String` | `eFetchRsInfoUrl` |
| `static java.lang.String` | `eFetchUrl` |
| `static java.lang.String` | `eLinkUrl` |
| `static java.lang.String` | `eSearchUrl` |
| `static java.lang.String` | `eUtilBase` |
| `static java.lang.String[]` | `genBankAcc` |
| `static java.lang.String[]` | `locusLinkId` |

**Fields inherited from class affychip.snpdetector.model.SnpDetector**

`findLocusLinkForAccessionNo, ncbiGeneAccessionNoSearchUrl, ncbiNucleotideDbUrl, ncbiRefSnpClusterUrl, ncbiSnpDbUrl`


**Constructor Summary**

`OptimizedSnpDetector()`


|  |  |
| --- | --- |
| **Method Summary** | |
| `void` | `detectSnpsFor(GeneInfo geneInfo)`             This method accesses a gene with a given accession number (from GenBank) and locus-link-id online and searches NCBI snp database for existence of SNPs in this gene's nucleotide-sequence. |
| `void` | `eFetch(java.lang.String refSeqId, GeneInfo geneInfo)`             This method accesses a gene with a given accession number (from GenBank) and locus-link-id online and searches NCBI snp database for existence of SNPs in this gene's nucleotide-sequence. |
| `java.lang.String` | `eLink(java.lang.String idList)` |
| `java.lang.String` | `eSearch(java.lang.String termsList)` |
| `static void` | `main(java.lang.String[] args)` |

**Methods inherited from class affychip.snpdetector.model.SnpDetector**

`dectectSnpsFor, findLocusLinkForAccessionNumber`

**Methods inherited from class java.lang.Object**

`clone, equals, finalize, getClass, hashCode, notify, notifyAll, toString, wait, wait, wait`

**Field Detail**

### eUtilBase

```
public static java.lang.String eUtilBase
```

---


### eSearchUrl

```
public static java.lang.String eSearchUrl
```

---


### eFetchUrl

```
public static java.lang.String eFetchUrl
```

---


### eLinkUrl

```
public static java.lang.String eLinkUrl
```

---


### eFetchRsInfoUrl

```
public static java.lang.String eFetchRsInfoUrl
```

---


### locusLinkId

```
public static java.lang.String[] locusLinkId
```

---


### genBankAcc

```
public static java.lang.String[] genBankAcc
```


**Constructor Detail**

### OptimizedSnpDetector

```
public OptimizedSnpDetector()
```


**Method Detail**

### detectSnpsFor

```
public void detectSnpsFor(GeneInfo geneInfo)
```

:   This method accesses a gene with a given accession number (from GenBank)
    and locus-link-id online and searches NCBI snp database for existence of
    SNPs in this gene's nucleotide-sequence. Both parameters are retrieved
    from the passed GeneInfo.

    :   **Overrides:**: `detectSnpsFor` in class `SnpDetector`
    :   **Parameters:**: `geneInfo` - type GeneInfo

---


### eSearch

```
public java.lang.String eSearch(java.lang.String termsList)
```

---


### eLink

```
public java.lang.String eLink(java.lang.String idList)
```

---


### eFetch

```
public void eFetch(java.lang.String refSeqId,
                   GeneInfo geneInfo)
```

:   This method accesses a gene with a given accession number (from GenBank)
    and locus-link-id online and searches NCBI snp database for existence of
    SNPs in this gene's nucleotide-sequence. Both parameters are retrieved
    from the passed GeneInfo.

    :   **Parameters:**: `geneInfo` - type GeneInfo

---


### main

```
public static void main(java.lang.String[] args)
```


---


|  |  |  |  |
| --- | --- | --- | --- |
| **Overview** \| **Package** \| **Class** \| **Use** \| **Tree** \| **Deprecated** \| **Index** \| **Help** | | |  |
| **PREV CLASS**   **NEXT CLASS** | **FRAMES**    **NO FRAMES**     **All Classes** |  |  |
| SUMMARY: NESTED \| FIELD \| CONSTR \| METHOD | DETAIL: FIELD \| CONSTR \| METHOD |  |  |


---
